# Mixed Multiple Autoimmune Syndrome Type 3 With Coexistence of Primary Biliary Cholangitis, Inflammatory Myopathy, and Chronic Thyroiditis: A Case Report

**DOI:** 10.7759/cureus.92751

**Published:** 2025-09-19

**Authors:** Bryan Nicolás Forero Vásquez, Carlos Daniel Henao Zuluaga, Alejandro Parra Peña, Eugenio Meek, Daniela Garcia Pereira

**Affiliations:** 1 Internal Medicine, Fundación Hospital San Carlos, Bogotá, COL; 2 Internal Medicine, Universidad Nacional de Colombia, Bogotá, COL; 3 Internal Medicine, Universidad del Rosario, Bogotá, COL; 4 Pathology, Fundación Hospital San Carlos, Bogotá, COL; 5 Internal Medicine, Hospital San Blas, Bogotá, COL

**Keywords:** autoimmune polyendocrinopathies, case report, hashimoto's disease, inflammatory myopathy, multiple autoimmune syndrome, primary biliary cholangitis

## Abstract

Multiple autoimmune syndromes (MAS) are a heterogeneous group of endocrine and non-endocrine autoimmune diseases. We present the case of a 78-year-old female with mixed type 3 MAS due to the presence of primary biliary cholangitis (PBC) and idiopathic inflammatory myopathy (IIM) with hypothyroidism due to Hashimoto's disease (HD), who was treated with corticosteroid pulses and azathioprine. Mixed MAS cases are rare, which implies the need for further studies to establish protocols for subclinical screening and management of simultaneous autoimmune pathologies with varying degrees of severity.

## Introduction

Multiple autoimmune syndrome (MAS), also termed autoimmune polyglandular syndrome (APS), is defined by the coexistence of two or more autoimmune diseases, most commonly endocrinopathies [1,2]. Classically, MAS is divided into four types [2]. Type 1 (MAS-1) comprises at least two of the following: chronic mucocutaneous candidiasis, chronic hypoparathyroidism, and Addison’s disease (AD), with autoimmune thyroid disease (AITD) variably present [2,3]. Type 2 (MAS-2) denotes AD in combination with AITD and/or type 1 diabetes mellitus [2,4]. Type 3 (MAS-3) is distinguished by AITD - either Hashimoto’s disease (HD) or Graves’ disease (GD) - together with one or more non-endocrine autoimmune conditions and, by definition, in the absence of AD [2]. Type 4 (MAS-4) encompasses associations of autoimmune diseases that do not fit the preceding categories [2].

We report a rare case of mixed MAS-3 in a patient with hypothyroidism due to HD, manifested by the coexistence of primary biliary cholangitis (PBC) and immune-mediated necrotizing myopathy, with a favorable response to corticosteroids and azathioprine.

## Case presentation

A 78-year-old woman from Boyacá, Colombia, was admitted to an outside hospital in May 2024 with 15 days of epigastric and right upper-quadrant pain, fatigue, generalized weakness, and diffuse jaundice. Initial laboratory tests showed elevated transaminases, direct hyperbilirubinemia, and an increased alkaline phosphatase. She had mild anemia with normal indices and a normal reticulocyte count. The direct Coombs test was negative; HIV was negative; Venereal Disease Research Laboratory (VDRL) was non-reactive; hepatitis B surface antigen was negative; and total hepatitis C antibodies were negative. Contrast-enhanced abdominal CT suggested cholecystitis, and ampicillin-sulbactam was started for presumed obstructive biliary syndrome secondary to cholecystitis.

**Figure 1:**
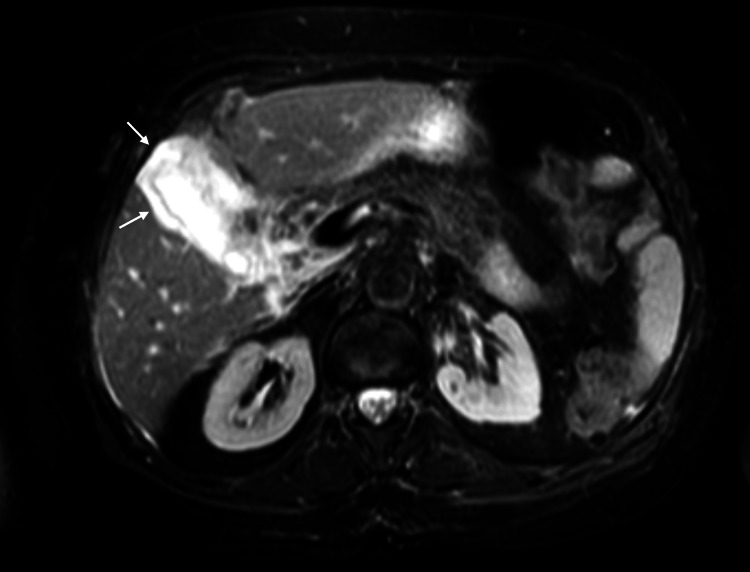
Axial T2-weighted fat-suppressed cholangioresonance showing diffuse gallbladder wall thickening (arrows) without intraluminal stones.

She was referred to our institution three days later. She additionally reported unquantified weight loss over six months, night sweats, anorexia, and nausea. Her history included hypertension (on losartan) and hypothyroidism (on levothyroxine). On examination she had generalized jaundice and right upper-quadrant tenderness. Admission studies reproduced the prior liver profile with markedly elevated gamma-glutamyl transferase (Table 1), normal renal function and electrolytes, and a blood count again showing mild anemia. Hepatitis A antibodies were negative. Magnetic resonance cholangiopancreatography (MRCP) demonstrated a distended gallbladder with diffuse wall thickening and a small amount of perivesicular fluid, without intra- or extrahepatic ductal dilation and without stones (Figure 1).

**Table 1:** Liver profile of the patient upon admission

Test	Results	Reference
Total bilirubin	9.6 mg/dL	0 - 1 mg/dL
Direct bilirubin	7.7 mg/dL	0 - 0.2 mg/dL
Indirect bilirubin	1.9 mg/dL	0 - 0.3 mg/dL
Alkaline phosphatase	1843 UI/L	32 - 92 mg/dL
Gamma-glutamyl transferase	1619 U/L	8 - 34 mg/dL
Alanine aminotransferase	1067 UI/L	0 - 33 mg/dL
Aspartate aminotransferase	1965 UI/L	10 - 42 mg/dL

She was admitted to the general surgery service for antibiotic therapy and serial monitoring of liver chemistries. Over two weeks, transaminases began to decline; however, bilirubin continued to rise at the expense of direct bilirubin. A contrast-enhanced abdominal MRI showed findings similar to the MRCP, tumor markers were negative, and the internal medicine service was consulted.

Given the mixed cholestatic-hepatocellular pattern and the absence of cholelithiasis, PBC was suspected. Ursodeoxycholic acid and prednisolone (1 mg/kg/day) were initiated, with a subsequent decrease in bilirubin. Autoimmune testing revealed positive antinuclear antibodies (ANA) with a mitochondrial pattern, positive anti-smooth muscle antibodies (ASMA), positive antimitochondrial antibodies (AMA), a positive extractable nuclear antigen (ENA) panel, and the remainder within normal limits (Table 2). One week later, PBC with severe liver injury was confirmed, and azathioprine 50 mg every 12 hours was added. Her clinical status and liver tests improved.

**Table 2:** Patient’s Immunological profile

Test	Results	Reference
C3	156 mg/dL	90 - 180 mg/dL
C4	36.6 mg/dL	100-40 mg/dL
Anti-DNA antibodies	Non-reactive	greater than 1/10
Antinuclear antibodies	1/640	greater than 1/40
Antineutrophil cytoplasmic antibodies	Non-reactive	greater than 1/20
Anti-smooth muscle antibodies	59.1 U/mL	Negative: less than 20 Weak positive: 20-30 Strong positive: greater than 30
Antiperoxidase antibodies	146 U/L	0 - 16 U/L
Total extractable antinuclear antibodies	35.5 U/mL	0 - 20 U/mL
Anti-RO, LA, RNP, and SM antibodies	Non-reactive	-

**Figure 2:**
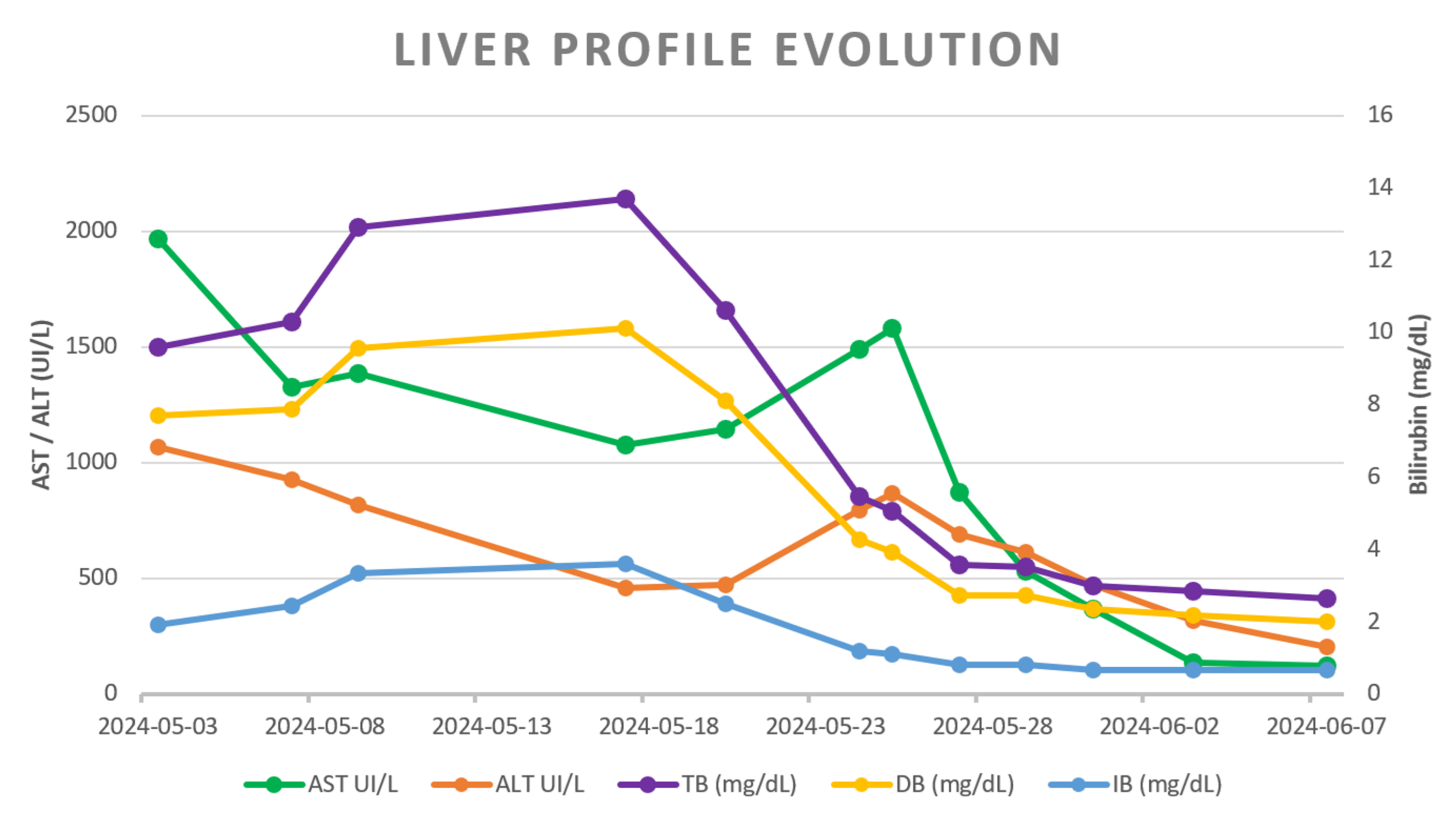
Liver profile evolution AST: Aspartate aminotransferase, ALT: Alanine aminotransferase, TB: Total bilirubine, DB: Direct bilirubine, IB: Indirect bilirubine.

Since admission, however, she had experienced progressive proximal lower-limb weakness that, over three weeks, rendered her unable to walk. Creatine phosphokinase was markedly elevated. Electromyography and nerve conduction studies demonstrated myopathic changes with intrinsic muscle fiber involvement, raising concern for idiopathic inflammatory myopathy (IIM) concomitant with PBC. An inflammatory myopathy antibody panel was negative. Because of the severity and persistence of weakness, methylprednisolone 500 mg/day was administered for three days, followed by prednisolone 1 mg/kg/day and physical therapy. Within one week, proximal weakness improved substantially, accompanied by near-complete resolution of direct hyperbilirubinemia and elevated transaminases (Figure 2).

**Figure 3:**
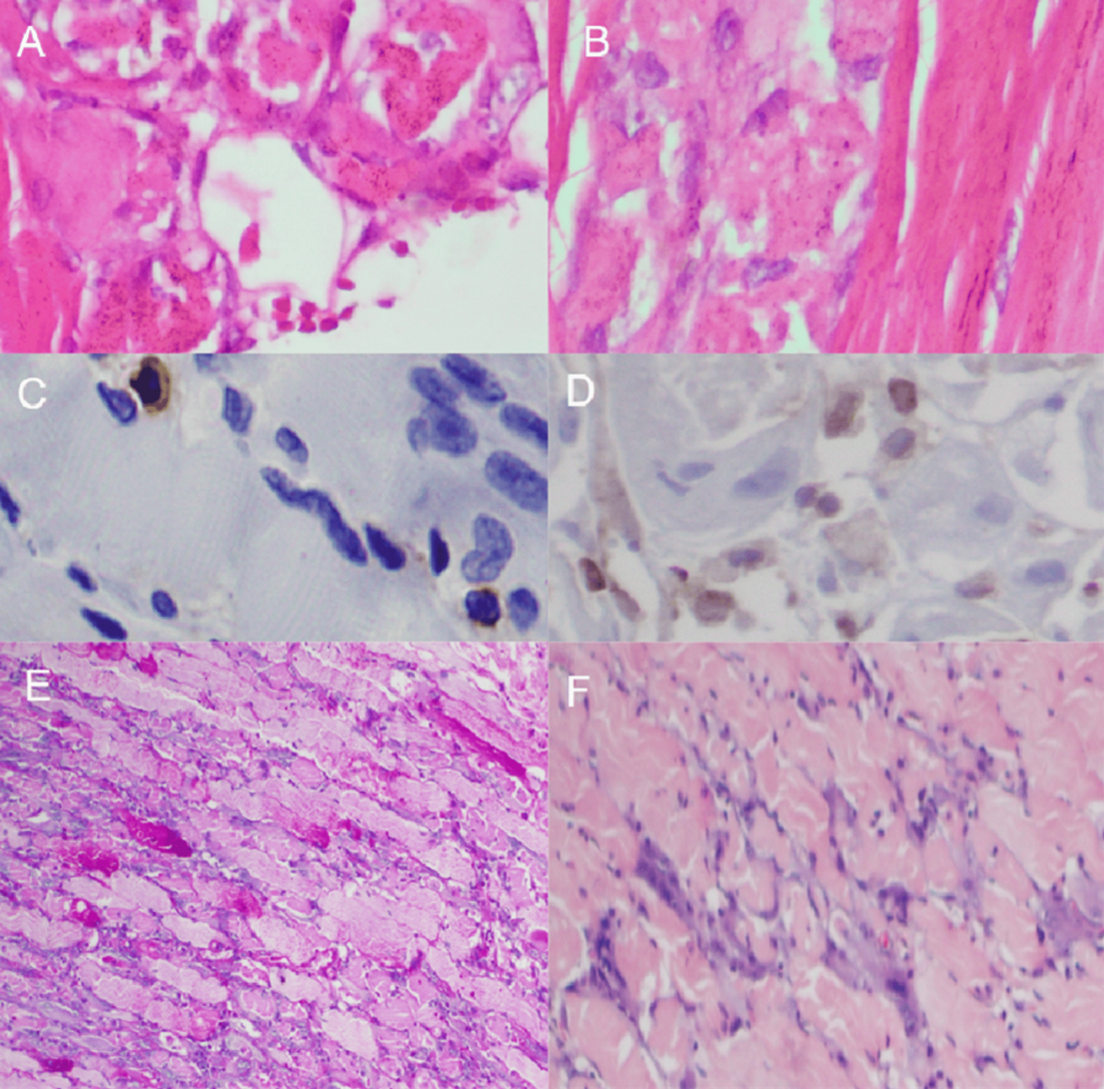
Histopathological findings from the deltoid muscle biopsy. Images A and B show several basophilic and necrotic myofibrils with phagocytosis, in the absence of endomysial inflammatory infiltrate, which is commonly seen in polymyositis and inclusion body myositis. There is evidence of minimal CD4 (C) and CD8 (D) inflammatory infiltrate, a finding consistent with immune-mediated inflammatory myopathy. Periodic acid-Schiff (PAS) staining in (E) highlighting atrophy in the fibers and in (F) trichrome staining with fibers undergoing degeneration.

Endoscopic ultrasonography showed a normal gallbladder and bile duct without wall thickening. A biopsy of the left deltoid muscle confirmed immune-mediated necrotizing myopathy (Figure 3).

A paraneoplastic process was excluded: upper endoscopy, colonoscopy, contrast-enhanced chest CT, head CT, and breast ultrasound were unremarkable. Thyroid ultrasound revealed a reduced, heterogeneously hypoechoic gland with thin hyperechoic septa, consistent with chronic thyroiditis; anti-thyroid peroxidase antibodies were positive.

In light of chronic autoimmune thyroiditis (Hashimoto’s disease), immune-mediated necrotizing myopathy, and PBC, she met criteria for MAS-3. During hospitalization, serial evaluations documented progressive clinical recovery with control of abdominal pain and sustained improvement in cholestasis (downtrending direct bilirubin) and hepatocellular injury (falling aminotransferases); creatine phosphokinase decreased in parallel, and proximal lower-limb strength improved with rehabilitation, permitting transition from bedbound to assisted ambulation by the time of discharge, she responded to corticosteroids and azathioprine and was discharged in good condition one month after admission. On outpatient follow-up, she achieved independent ambulation without assistance and had near-complete resolution of jaundice and she remained on ursodeoxycholic acid and azathioprine with gradual tapering of prednisolone, without relapse or treatment-related adverse events.

## Discussion

MAS is a rare, heterogeneous constellation of autoimmune diseases characterized by immune-mediated damage and inflammation affecting multiple organs, often including endocrine glands [5]. Within this spectrum, MAS-3 denotes autoimmune involvement of the thyroid - classically chronic thyroiditis due to Hashimoto’s disease or hyperthyroidism due to Graves’ disease - together with at least one non-endocrine autoimmune disease, in the absence of Addison’s disease [2,6]. The prevalence of MAS varies by type: type 1 is estimated at one per 100,000 people, whereas types 2-4 occur in approximately one per 20,000, with notable population-level variability [7].

Regarding classification, the widely adopted framework for polyendocrinopathies, including MAS-3, proposed by Betterle and Presotto in 2008 remains the foundation for current diagnostic groupings [4]. Building on that schema, MAS-3 subtypes were updated in 2019 to the now familiar A, B, C, and D categories, which better capture the phenotypic diversity observed in clinical practice [3,6].

Notably, the term “mixed MAS/APS” (overlap between multiple autoimmune syndrome and autoimmune polyglandular syndromes) has been reported only twice: first, in an adult with PBC, psoriasis, and vitiligo-classified as MAS-3B and MAS-3C; and second, in a pediatric patient with two endocrinopathies and eight non-endocrine autoimmune conditions, exhibiting features of types 1, 2, and 3 and thus described as a mixed phenotype [5,8].

**Figure 4:**
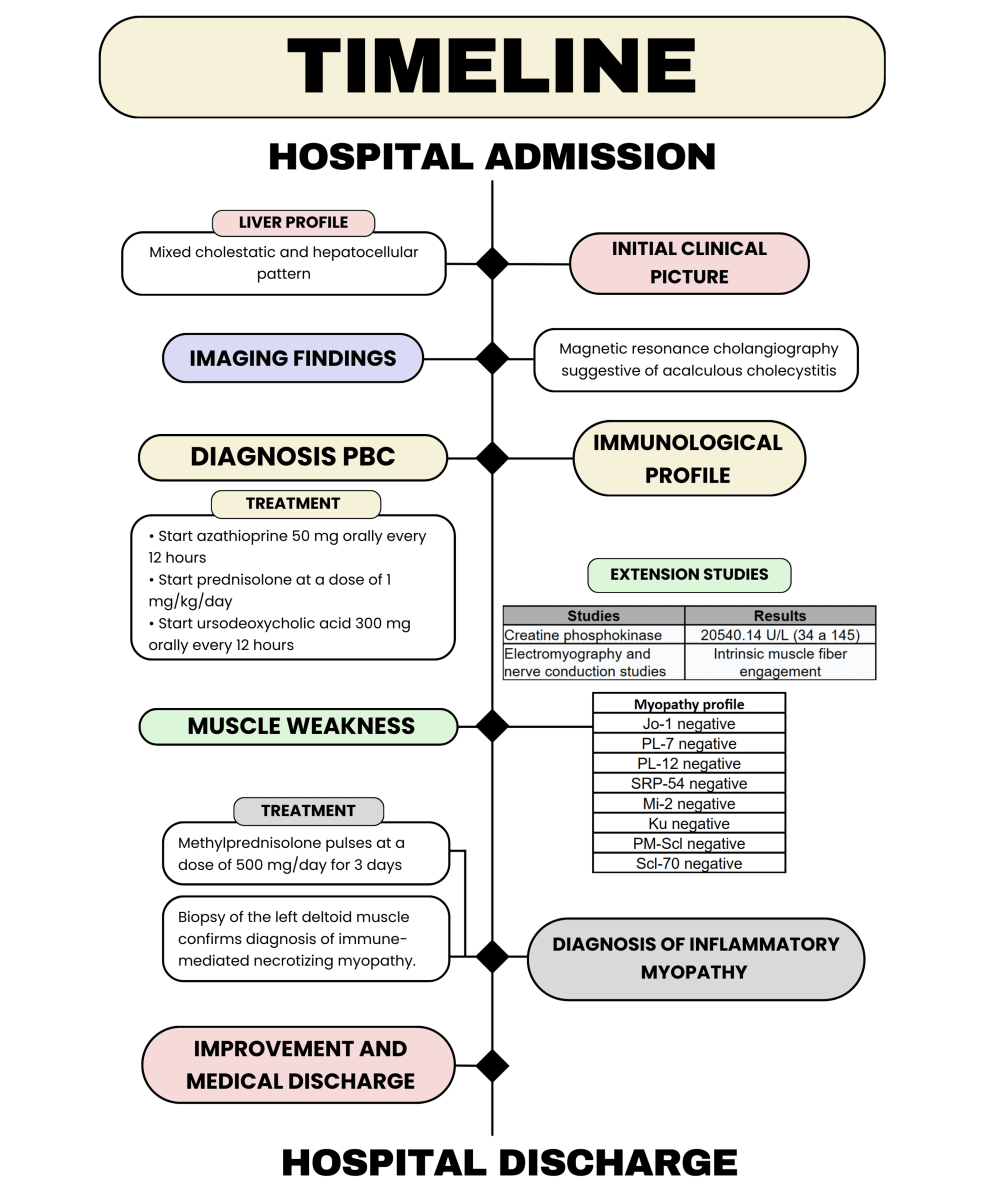
Timeline of the case report from admission to discharge from hospital. PBC: primary biliary cholangitis

Our case aligns with a mixed MAS-3 presentation, specifically PBC (subtype 3B) together with IIM (subtype 3D), consistent with the complete clinical and laboratory profile (see Figure 4). Contextually, autoimmune thyroid disease is not uncommon in PBC: a European series spanning 1975-2015 reported a 12% prevalence [9]. Likewise, thyroid disorders have been documented in patients with polymyositis and dermatomyositis, with rates ranging from 12% to 25% [10]. To date, we have found no studies specifically linking immune-mediated necrotizing myopathy to underlying thyroid disease; however, as a member of the IIM group, it is appropriately categorized under MAS-3D.

Beyond isolated associations, a PBC-IIM syndrome with positive AMA has been described in the literature, with reported prevalence ranging from 0.08% to 3.3% [11-13]. Nevertheless, current evidence does not clearly establish distinct therapeutic or prognostic implications for this overlap compared with the management and outcomes of the individual component diseases [14]. Accordingly, our case adds to the limited body of evidence supporting the recognition of mixed MAS-3 phenotypes and underscores the need for longitudinal studies to clarify optimal therapeutic strategies and long-term prognosis in these patients.

## Conclusions

Our case can be considered one of the few reported cases of a mixed form of MAS-3, with the rare association of CBP and IIM in HD. Although the pharmacological management of both entities is similar, in this patient it was necessary to administer corticosteroid pulses due to the greater severity of the muscular involvement compared to the hepatobiliary involvement, which was already showing improvement before the start of treatment. This scenario highlights the importance of physicians considering the possibility of overlap of more than one entity at the time of clinical evaluation, allowing for timely therapy. Therefore, studies are needed to define the therapeutic and prognostic implications so that protocols and tools can be established in the future to improve the identification of this entity.
